# An Abraded Surface of Doxorubicin-Loaded Surfactant-Containing Drug Delivery Systems Effectively Reduces the Survival of Carcinoma Cells

**DOI:** 10.3390/biomedicines4030022

**Published:** 2016-09-15

**Authors:** Christian Schmidt, Fabiano Yokaichiya, Nurdan Doğangüzel, Margareth K. K. Dias Franco, Leide P. Cavalcanti, Mark A. Brown, Melissa I. Alkschbirs, Daniele R. de Araujo, Mont Kumpugdee-Vollrath, Joachim Storsberg

**Affiliations:** 1 Department of Biomaterials and Healthcare, Division of Life Science and Bioprocesses, Fraunhofer Institute for Applied Polymer Research (IAP), Geiselbergstraße 69, 14476 Potsdam-Golm, Germany; christian.schmidt@iap.fraunhofer.de (C.S.); nurdandg@hotmail.de (N.D.); 2 Institute of Materials and Energy, Helmholtz-Center Berlin, Hahn-Meitner-Platz 1, 14109 Berlin, Germany; fabiano.yokaichiya@gmail.com; 3 Department of Pharmaceutical Engineering, Beuth University of Applied Sciences Berlin, 13353 Berlin, Germany; vollrath@beuth-hochschule.de; 4 Instituto de Pesquisas Energéticas e Nucleares, Avenida Lineo Prestes, 2342, Cidade Universitária Armando Salles de Oliveira, SP 05508-900, Brazil; mkfranco@ipen.br; 5 School of Chemical Engineering, University of Campinas, Campinas, SP 13083-970, Brazil; leide.p.cavalcanti@gmail.com; 6 Department of Clinical Sciences, Cell and Molecular Biology Program and Flint Cancer Center, Colorado State University, Fort Collins, CO 80523-1052, USA; M.Brown@colostate.edu; 7 Instituto de Química, Campinas, Universidade Estadual de Campinas, Campinas, SP 13083-970, Brazil; mel_inger@yahoo.com.br; 8 Centro de Ciências Naturais e Humanas, Universidade Federal do ABC, Santo André, SP 09210-580, Brazil; daniele.araujo@ufabc.edu.br

**Keywords:** drug delivery, doxorubicin, cancer therapy, synchrotron small-angle X-ray scattering, in vitro tests

## Abstract

An effective antitumor remedy is yet to be developed. All previous approaches for a targeted delivery of anticancer medicine have relied on trial and error. The goal of this study was to use structural insights gained from the study of delivery systems and malignant cells to provide for a systematic approach to the development of next-generation drugs. We used doxorubicin (Dox) liposomal formulations. We assayed for cytotoxicity via the electrical current exclusion method. Dialysis of the samples yielded information about their drug release profiles. Information about the surface of the delivery systems was obtained through synchrotron small-angle X-ray scattering (SAXS) measurements. SAXS measurements revealed that Dox-loading yielded an abraded surface of our Dox liposomal formulation containing soybean oil, which also correlated with an effective reduction of the survival of carcinoma cells. Furthermore, a dialysis assay revealed that a higher burst of Dox was released from soybean oil-containing preparations within the first five hours. We conclude from our results that an abraded surface of Dox-loaded drug delivery system increases their efficacy. The apparent match between surface geometry of drug delivery systems and target cells is suggested as a steppingstone for refined development of drug delivery systems. This is the first study to provide a systematic approach to developing next-generation drug carrier systems using structural insights to guide the development of next-generation drug delivery systems with increased efficacy and reduced side effects.

## 1. Introduction

Cancerous lesions are predicted to be the leading cause of deaths in the U.S. within the next five years [1]. Approaches to the clinical management of cancers include application of pharmacologically active substances which, in addition to the desired cytotoxic impacts upon tumorigenic cells, are often accompanied by adverse effects [2,3].

Reducing the unintended, chemotherapy-associated toxicity profile for cancer patients requires the development of new therapeutic formulations. One approach to address this unmet clinical need is the development of targeted drug delivery using carrier systems with a long shelf life, optimal drug-loading profile, and low inherent toxicity. See [4,5] for recent reviews.

Doxorubicin (Dox), either alone [6,7,8] or in combination with other treatment options [9], is used in the management of a wide range of cancerous lesions. Mechanistic insights into the p21-mediated negative influence of Dox on the cell cycle were provided in 2011 [10]. In 2014, its cardiac toxicity was reported to involve mitochondrial accumulation of iron [11].

Phase III clinical trials concluded that the cardiac toxicity of liposomal formulated Dox was reduced while efficacy remained comparable to conventional Dox [12]. On 4 February 2013, shortage of the brand Doxil^®^ prompted the U.S. Food and Drug Administration to approve the usage of a generic version of this liposomal Dox formulation for the treatment of, e.g., advanced or persistent ovarian cancer after prior platinum-derived chemotherapy [13].

While cardiotoxicity associated with liposomal Dox formulation appears to be reduced, there is an apparent correlation between the liposomal formulation used in the clinical setting and incidents of hand-and-foot-syndrome (HFS). Other incidents of HFS and formulation of chemotherapeutic agents are not formally established. Further research is necessary to address the underlying concerns voiced in published evidence [14,15].

The use of lipid-based colloidal carriers is widely known, like administration of lipophilic drugs and reduction of local toxicity, among others. These carrier systems offer the possibility of modulating drug release because they facilitate the drug transport to different biological tissues, to tumor and inflammatory cells, and to a large variety of organs, like liver, lung, and heart. Moreover, they increase the local penetration, extending residence time, and they control release mechanism in order to supply an effective dose to the target site. Our research will be focus on two lipid systems: soybean oil and Mygliol 812.

The existence of fractal surfaces in human biology is not without precedence. For instance, the Bowman’s membrane of the human cornea is ascribed to display a fractal surface [16]. Furthermore, the suggestion of clot fractality as a biomarker for different stages of lung cancer adds further credence to this notion [17]. The third line of support comes from a report where atomic force microscopy (AFM) analysis revealed a correlation of fractal geometry of cells with the transition from immortality to tumor [18].

Here, we aimed at correlating the efficacy of drug delivery systems with surface characteristics. In order to show this correlation, we have performed small angle X-ray scattering (SAXS) measurements in the drug delivery systems, soybean oil and Mygliol 812, with and without Dox, and in vitro tests with HeLa and HCT116 cells.

## 2. Experimental Section

### 2.1. Materials

The following reagents were obtained from the following vendors: 2,6-di-tert-butyl-4-methylphenol (BHT, ACROS, Geel, Belgium, Lot # A0203142), soybean oil (Roth, Karlsruhe, Germany, Lot # 352187856), cetyl palmitate (Roth; Lot # 452162569), Sodium tetradecyl sulfate (Alrdich, Munich, Germany, Lot # MKBK1230V), Mygliol 812 (Caesar & Loretz, Hilden, Germany, Lot # 12078605), polysorbate 80 (Roth, Lot # 292187273 and 302187273), 1,2-dimyristoyl-sn-glycero-3-phosphocholine (l-α-lecithin; Roth, Lot # 312189810) and doxorubicin (Molecula, Munich, Germany, Lot # 201249 and Sigma, Munich, Germany, Lot # SLBC8638V). The composition of soybean oil and Mygliol 812 used in this study is detailed in Table 1 and Table 2, respectively. Soybean oil is composed of about 86% of unsaturated fatty acids whereas Mygliol 812 is composed of 99% of saturated fatty acids.

### 2.2. Carrier Preparation

For carrier preparation, we followed [19] with modifications: the aqueous phase consisted of 15.2 g water (1.84 mM [Dox] for loading) and 2 g polysorbate 80, whereas the lipid phase consisted of 300 mg lecithin, 500 mg BHT, 3.75 g cetyl palmitate, and either 3.75 g soybean oil or Mygliol 812.

Individual containers were used to preheat both mixtures to 70 °C under constant agitation at 600 rpm. The aqueous phase was then added dropwise to the lipid phase under constant agitation (600 rpm) at 70 °C. As preparation for the sonication step, we continued agitation at 70 °C for five minutes, removed the heat source and continued agitation until the mixture reached room temperature (25 °C). Five individual sonication steps for 5 min each with a 10 s pause took place using the following settings: 40% amplitude on a Sonoplus Mini 20 from Brandt Electronics, Milpitas, CA, USA. After reaching the desired pH 8 ± 0.1, we passed the product through 1 µm and 0.45 µm membrane filters, successively, and added sodium tetradecyl sulfate (STS) at a molar ratio of 1.25 mol STS per 1 mol Dox, followed by refrigeration for 3 days and sonication (3 min; 40% amplitude on a Sonoplus Mini 20 from Brandt Electronics).

### 2.3. Particle Analysis

We examined mean hydrodynamic particle size and zeta (ζ) potential using samples diluted 1:2500 in particle-free water. To assay for the ζ-potential, we diluted 2.5 µL carrier formulation in 10 mL particle-free ultrapure water. Using water as a reference, measurements were carried out using the Zetasizer 3000 (Malvern Instruments, Northampton, MA, USA). Using the same dilution regimen, we determined the distribution of particle sizes using a high performance particle size reader (Malvern Instruments).

### 2.4. Tissue Culture

Tissue culture plastic was obtained from TPP, Trasadingen, Switzerland. Biochrom AG, Berlin, Germany was chosen as supplier for Dulbecco’s Modified Eagle’s Medium (DMEM), phosphate-buffered saline (PBS without Mg^2+^/Ca^2+^), Roswell Park Memorial Institute Medium (RPMI) 1640, fetal bovine serum (FBS), ultrapure water, 10× stock solution of trypsin/ethylenediaminetetraacetic acid (EDTA), and 100× stock solutions of sodium pyruvate, nonessential amino acids, and penicillin/streptomycin. Dr. Norbert Nass (Otto-von-Guericke-University, Institute of Pathology, Magdeburg, Germany) graciously gifted us with HeLa and HCT116 cells. Cells were maintained in RPMI-1640, supplemented to a final concentration of 10% heat-inactivated FBS, 4 mM l-glutamine, 2 mM sodium pyruvate, and 500 U penicillin and 500 U streptomycin. Subcultivation of the cells occurred after the monolayer reached 75%–80% confluency [19]. After rinsing adherent cells with PBS, incubation with 13 µL/cm^2^ culture area trypsin/EDTA followed for 5 min at 37 °C with gentle knocking of the tissue culture flask until cells started to detach. Trypsin/EDTA was inactivated by adding complete growth medium and washing detached cells from the culture area followed by refreshment of culture medium and a count of cells. Appropriate cell densities were then reached by dilution of the suspension in growth medium and plating cells onto a fresh culture area.

### 2.5. In Vitro Tests

For the in vitro performance test of synthesized carrier systems on HeLa cells, we seeded 10^4^ HeLa cells per cm^2^ culture area on day −3 into a well of a 12-well plate and grew them for 3 days (until day 0) in 1 mL of growth medium. On day 0, growth medium was replenished and supplemented with either Dox from a 10 mg/mL stock solution in growth medium, or either (1) loaded carrier system to deliver the amounts of Dox indicated in the figures or (2) comparable amounts of unloaded carriers. Viable and dead cells were determined after incubation for 3 days (day 3) as described above. For tests involving HCT116 cells, we seeded 2.5 × 10^3^ HCT116 cells per cm^2^ of culture area on day −2 into a well of a 12-well plate and grew them for 2 days (until day 0) in growth medium. On day 0, growth medium was replenished and supplemented as described above. Viable and dead cells were determined after incubation for 2 days (day 2) as described above. The relative survival rate of the cells, measured via the electrical resistance of cells in the Casy TT system (Roche, Mannheim, Germany) using growth medium as reference, is plotted in reference to untreated cells (set to 100%). All experiments were performed in triplicates with mean values and standard deviations plotted using Microsoft Excel^®^.

### 2.6. Small-Angle X-ray Scattering (SAXS)

SAXS measurements were performed at room temperature (22 °C) at the Brazilian Synchrotron Light Laboratory (LNLS) using the SAXS2 beamline with an energy of 8 keV (λ = 1.5498 Å), wave vector numbers ranging from 0.25 to 6.5 nm^−1^, 1 m distance between sample and the MarCCD detector (diameter of 165 mm) and a customized sample holder [20]. Samples were placed into a 600 µm diameter channel to obtain a sample thickness of 1 mm between replaceable mica windows.

Usage of this device ensures a constant position of the sample in reference to the beam path. By measuring a buffer solution sample or water sample, which has a scattering curve that is a constant line, we monitored the background scattering and subtracted this intensity curve from the actual sample. Samples were retrieved after data acquisition and the cell was rinsed and dried using nitrogen gas. The background-corrected and normalized scattering intensity was recorded by a 2-dimensional detector (MAR-Rayonix, Evanston, IL, USA, 165 mm diameter). In the case of isotropic scattering, the beam was set to target the center of the detector. Integration of the area over the solid angle was then used to determine the dependency of the isotropic intensity as a function of the scattering angle [20].

## 3. Results

### 3.1. Synthesis and Characterization of Carrier Systems

**Figure 1:**
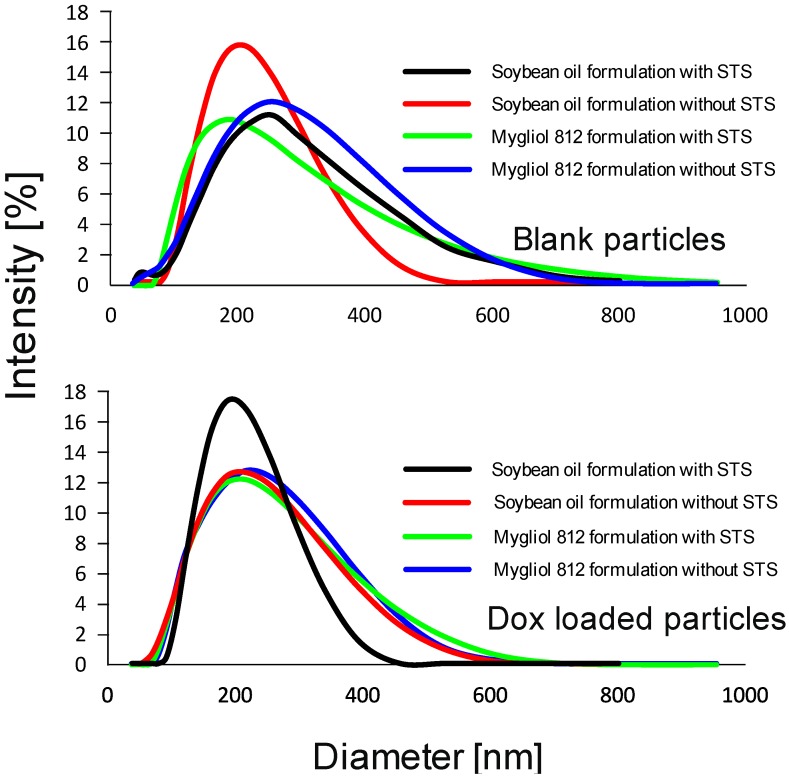
Freshly prepared carrier systems display a peak hydrodynamic particle size of approximately 200 nm. Freshly prepared particles (blank: top panel; doxorubicin (Dox)-loaded particles: bottom panel) were diluted 1:2500 in particle-free water and measured using a Malvern particle size reader. The distribution of the hydrodynamic particle size (in nm) is plotted against the intensity (in %). Individual preparations are denoted by black: soybean oil formulation with sodium tetradecyl sulfate (STS); red: soybean oil formulation without STS; green: Mygliol 812 formulations with STS; blue: Mygliol 812 formulations without STS.

At first, we assayed for mean hydrodynamic particle size distribution, as described in [21] and above, using freshly prepared material (Figure 1).

Briefly, we diluted the formulation 1:2500 in particle-free water using a Malvern particle size reader and found a unimodal distribution of the dynamic particle size with an approximately 200 nm peak (Figure 1). Our analyses revealed that the size distribution of the particles was not uniform if different compositions are interrogated (Figure 1). As for blank particles, graphs for soybean-based materials increases, whereas the opposite phenomenon is seen for Mygliol-based carrier formulations. In both cases, the form of the curve changes as well; this is most obvious in the case of soybean-based carriers, where the width of the curve increases toward particles with larger sizes. Looking at soybean-based carriers that are loaded with drug, the observation seems to be diametrically opposite: the width of the curve decreases. This observation deems to be less pronounced in the case of Mygliol-based formulations where the respective graphs can be approximated as overlaps (Figure 1). In general, there is precedent in the literature for a decreased particle size of carrier systems after loading with Dox [22,23]. We conclude from these observations, in agreement with published studies, that the carrier systems were successfully loaded with the drug.

**Figure 2:**
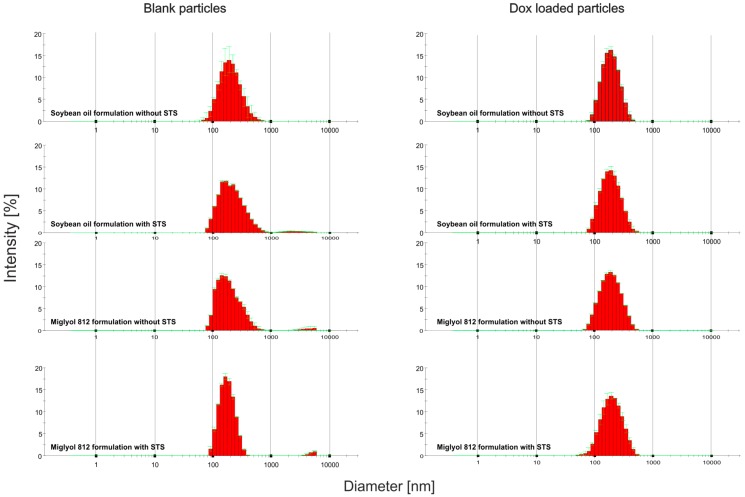
Stable mean hydrodynamic particle size distribution of delivery systems after 7 months of storage at 4 °C in the dark. The same preparations that were measured in Figure 1 were stored for 7 months in the dark at 4 °C and subjected to a determination of the mean hydrodynamic particle size distribution, as described in Figure 1 and above. The distribution of the hydrodynamic particle size (in nm) is plotted against the intensity (in %). Individual preparations are denoted on the left side of each graph.

To address the susceptibility of the particles to Ostwald ripening, we assayed for distribution of the hydrodynamic particle size after 7 months of storage at 4 °C in the dark [24,25]. There were no profound changes in the mean hydrodynamic particle size distribution of stored particles in comparison to freshly prepared materials (Figure 1 and Figure 2).

To provide additional data in support of our notion that the loading of the particles was successful in addition to the above-described altered particle size (see above), we then assayed for the ζ-potential of plain and Dox-loaded carrier systems because there is precedent in the literature for a reduced ζ-potential in response to loading with Dox [26]. We observed a decrease (absolute values) of 14.44 mV for the soybean-based formulation (loaded: −37.92 ± 0.86 mV; plain: −52.36 ± 0.34 mV) and 7.96 mV for Mygliol 812-based carriers (loaded: −27.98 ± 0.92 mV; plain: −35.54 ± 0.26 mV). Our observed reduced negativity of the ζ-potential after loading with Dox ties in nicely with Jain et al. [27], although the differences in absolute reduction, 21.81 mV in [26] versus our observations of ~14 mV and 8 mV for soybean- or Mygliol 812-based preparations, respectively, may provide credence for the notion for a material-specific reduction of the negative ζ-potential in Dox-loaded carrier systems.

To further describe the carrier formulations obtained with regard to the speed of drug release [28], we employed a dialysis assay assayed for the release of Dox. For this, we placed 300 µL of the indicated drug carriers (equal amounts of Dox-loaded) in a dialysis bag (Mw cut off: 4–6 Da; thickness: 28 µm; nominal filter rate of 3.5; obtained from Roth) and dialyzed this against a volume of 50 mL buffer (pH 7.4), consisting of 10 vol.% ethanol, 15 vol.% polysorbate 80, and 70 vol.% Sørensen’s buffer under constant agitation (600 rpm) with 2 mL of medium drawn at 1, 2, 4, 7, 24, 32, and 48 h to determine the amount of Dox by comparing the optical density of a reference to the sample.

**Figure 3:**
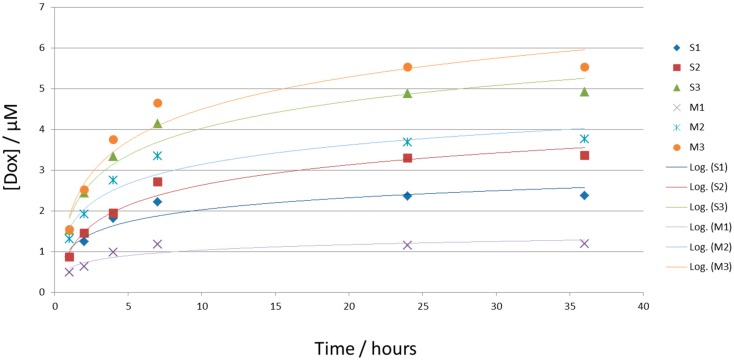
Uniform release characteristics of carrier systems tested. Equal amounts of Dox-loaded carriers were placed in a dialysis bag and dialyzed against a volume of 50 mL buffer under constant agitation (100 rpm). Optical densities of samples, corrected against dialysis medium were used to determine [Dox].

Plotting [Dox] in µM against time in hours resulted in the graph shown in Figure 3. Equal amounts of Dox-loaded carriers (300 µL) were placed in a dialysis bag and dialyzed against a volume of 50 mL buffer (pH 7.4; consisting of 10 vol.% ethanol, 15 vol.% polysorbate 80, and 70 vol.% Sørensen’s buffer) under constant agitation (100 rpm). At time points 1, 2, 4, 7, 24, and 36 h, 500 µL were drawn to determine the amount of Dox by comparing the optical density of a reference to the sample. We plotted [Dox] in µM against time (in hours). Values obtained using soybean oil- or Mygliol-based carriers are graphed on the left-hand panel including a log-style trend curve with S0 and M0 denoting plain formulations, and Dox-loaded carriers denoted as M1 containing 180 µM; S1 containing 360 µM; M2 and S2 containing 540 µM; and M3 and S3 each containing 900 µM Dox. Our observations indicate a burst of Dox release until 7 h, followed by a plateau phase after 24 h in all carrier systems tested. All carriers displayed similar curve characteristics [28]. We conclude that the release of Dox over time is independent of the composition of the carrier system.

### 3.2. In Vitro Performance of Synthesized Carrier Systems on HeLa and HCT116 Cells

In the in vitro tests, we used the differential sensitivity of HeLa and HCT116 cells to chemotherapeutic drugs, such as Dox, ethyl methane-sulfonate, and 5-fluorouracil [28,29]. We plotted the medium-corrected, relative survival rate of either HeLa or HCT116 cells in response to Dox solution, blank, or loaded drug delivery and accounted for possible cytotoxic properties of empty carrier systems by adding comparable amounts of empty carriers as were needed to the delivery of the concentration of Dox in the indicated treatments with loaded nanoparticles.

**Figure 4:**
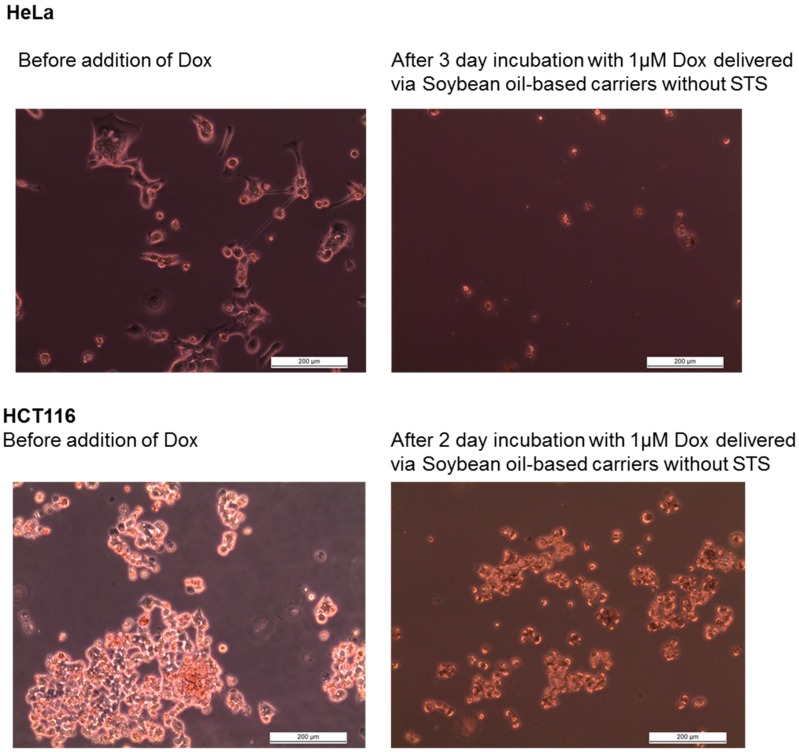
The viability of HeLa and HCT-116 cells is reduced after exposure to Dox soybean oil-based carrier systems. Shown here are phase contrast images with focus on adherent cells of HeLa (top panel) and HCT116 cultures (bottom panel) before and after exposure to 1 µM of Dox delivered via soybean oil-based carriers without STS. These images were taken before cells were harvested for the determination of the survival using the Casy TT system, as described in the Methods section. Note the changed morphology in response of the treatment with Dox. The results of the quantification are shown in Figure 5.

**Figure 5:**
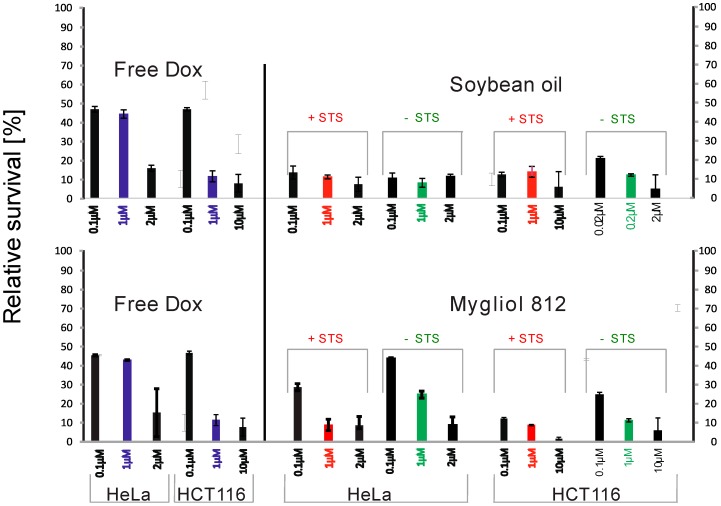
The survival of HeLa cells is reduced the strongest with Dox-loaded and STS-containing soybean oil-based carrier systems. Shown here are medium-corrected, relative survival rates of either HeLa or HCT116 cells in response to Dox. To account for the eventuality of cytotoxic properties of empty carrier systems, we added comparable amounts of empty carriers as were needed to the delivery of the concentration of Dox (not shown). Red bars indicate 1 µM free Dox, and violet bars indicate 1 µM Dox delivered via carrier systems. The results for soybean oil-derived formulations are shown in the top panel, whereas and the results for the corresponding Mygliol 812-derived formulations are shown in the bottom panel.

In our assays, using a pharmacologically relevant [Dox] of 1 µM [28] yielded a constant survival rate of HeLa cells, whereas the survival of HCT116 cells was reduced roughly 5-fold under the same conditions (Figure 4 and Figure 5). Delivery of 0.1 µM Dox with soybean oil-based carriers reduced the survival 3- and nearly 5-fold for HeLa and HCT116 cells, respectively, whereas Mygliol 812-based drug carriers proved to be less effective in reducing the survival rate of either HeLa or HCT cells (decrease by 2-fold at 1 µM Dox for HeLa and 2-fold at 0.1 µM Dox for HCT116; all in comparison to free Dox in growth medium; Figure 5). Presence of STS in soybean oil-based carrier systems reduced the survival rate of both HeLa and HCT cells by a factor of 5, with comparable Mygliol 812-based drug carriers reducing the survival rate of HeLa cells by a factor of 2 (at 1 µM Dox) and a factor of 5 for HCT116 cells (at 0.1 µM Dox; all in comparison to free Dox in growth medium; Figure 5). The survival of HeLa cells is reduced the strongest with Dox-loaded and STS-containing soybean oil-based carrier systems in comparison to their respective counterparts.

### 3.3. SAXS Characterization

In vitro performance assays using used plain carriers, denoted as S0 and M0, and Dox-loaded carriers (M1: 180 µM and S1: 360 µM) yielded potent effects of STS-containing and Dox-loaded soybean (S) oil-based carrier systems in comparison to their respective Mygliol 812 (M) counterpart (see above). We therefore wished to obtain structural insights into these carrier systems to possibly correlate them with our in vitro performance results. Therefore, SAXS beamline measurements were performed at the Brazilian Synchrotron Light Laboratories as described above. We used plain carriers, denoted as S0 and M0, and Dox-loaded carriers (M1: 180 µM; S1: 360 µM; M2 and S2: 540 µM; M3 and S3: 900 µM) in our measurements.

**Figure 6:**
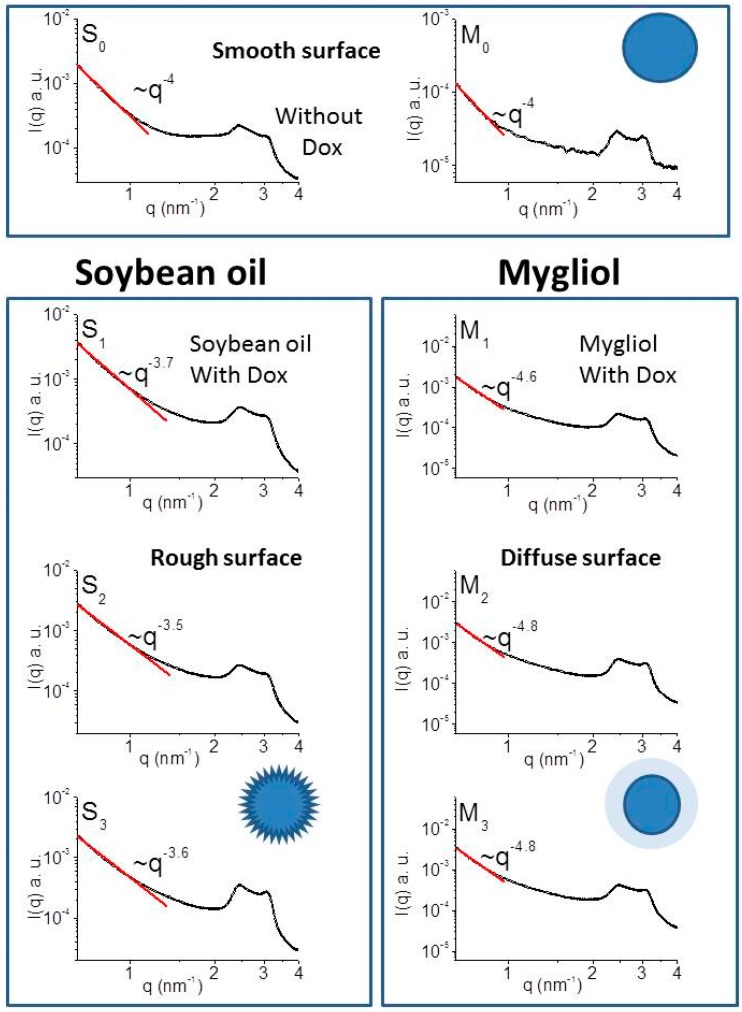
Loading with Dox abrades the surfaces of STS-containing soybean oil carriers. Shown here are the results of SAXS measurements using STS-containing Mygliol 812 (M) and Soybean (S) oil-based carriers with S0 and M0 denoting plain formulations, and Dox-loaded carriers labeled with the amounts of Dox loaded (M1: 180 µM; S1: 360 µM; M2 and S2: 540 µM; M3 and S3: 900 µM). The intensity of the scattering event I is plotted using arbitrary units against the scattering vector q in nm^−1^ with the approximated tangent of the graph denoted as red line in the respective plots. In addition, a graphical interpretation of the results obtained is provided. Both plain soybean oil and plain Mygliol 812-based systems display a smooth surface in accordance with [30]. Loading with Dox, regardless of the amounts loaded, roughens the surface in the case of soybean oil carriers. In stark contrast, Mygliol 812-based formulations yield a diffuse surface after loading with Dox.

As shown in Figure 6, we observed two peaks at positions q1 = 2.50 nm^−1^ and q2 = 3.02 nm^−1^, implying that all the samples presents 3-dimensional cubic structure [30]. The slope |α| of for small q was determined to be: 4 for blank carriers, 3.7 for S1, 3.5 for S2, and 3.6 for S3. As for the systems M1, M2, and M3, the respective |α| values are 4.6, 4.8, and 4.8 (Figure 6). These values are indicative of an alternated surface geometry after Dox loading of our carrier systems.

## 4. Discussion

In this study, we report the synthesis of soybean oil- or Mygliol 812-based drug delivery systems with an average hydrodynamic diameter of 200 nm, a shelf life of at least 7 months when refrigerated, and a reduced negativity of the ζ-potential in response to loading with Dox. Furthermore, we found that a greater burst of Dox is released from soybean oil-based formulations, which ties in nicely with an elevated efficacy of Dox-loaded carriers to reduce the viability of HeLa cells at a pharmacologically relevant [Dox] of 1 µM when STS is present in these formulations. Structural interrogation of these systems revealed that loading with Dox abraded the initially smooth surface of soybean oil-based carrier formulations, in stark contrast to a diffused surface of Mygliol 812-based formulations under comparable conditions.

Our SAXS experimentation revealed support for the notion that the Dox-loaded and STS-containing soybean oil-derived or Mygliol 812 drug delivery systems are indirectly connected to the surface of the system with a dense core regardless of the amounts of Dox loaded. The probability of the formation of hybrid electron density distributions as a result of the scattering event itself allows for estimations of absolute values of the slope α of the intensity of the scattering signal I [30]. There is precedent in the literature for correlations of |α| with surface characteristics: For instance, 3 < |α| < 4 is ascribed to indicate materials with a rough surface and a dense core. A uniform density and smooth surface is apparently indicated if |α| = 4, whereas |α| > 4 is used to indicate materials with a diffuse (or fuzzy) surface with varying density [30].

Therefore, for the STS-containing soybean oil-derived system, the value of the power law scattering exponent |α| demonstrates a fractal surface which explains a better interaction with the fractal surface of HeLa cells [18]. Likewise, for STS-containing Mygliol 812-derived drug delivery systems, |α| indicates that the surface of the system has a diffuse or fuzzy nature, indicating that the interface region varies gradually over a short distance through the inner part of the particle. Consequently, the interaction with the cancer cell shows a low efficiency as proved in our in vitro results. Moreover, the SAXS results suggest that, comparing the fatty acid compositions of both systems, soybean oil and Mygliol 812, the Dox interacts in a different way with unsaturated and saturated fatty acid. In the case of Mygliol 812, where more than 99% are unsaturated fatty acid, as indicated in the Materials section, Dox changes the systems from smooth surface to fuzzy surface. On the other hand, for soybean oil, where more than 86% are composed with saturated fatty acid, Dox changes the system from smooth surface to fractal surface. Credence for the notion of an apparent match between the surface geometry of HeLa cells and the soybean oil drug delivery systems is provided by our observation of reduced viability of HeLa cells after exposure to 0.1 µM Dox delivered by soybean oil-based carriers in comparison to their respective Mygliol 812 counterparts.

The carrier systems surface was abraded after Dox loading of soybean oil carriers. Further studies are necessary to explicate the different surface geometry of the carrier systems studied. The analysis obtained by SAXS consolidates the results observed in vitro using HeLa and HCT116 cells. The SAXS data analysis shows that the interaction of the drug in the rough surface (soybean oil) provides a better delivery when compared to the interaction of the drug in the diffuse surface (Mygliol 812).

In conclusion, this is the first study where characteristics of the surface geometry of drug delivery systems, expressed as fractality, is correlated with their antitumor efficacy based on compounds used in food and cosmetic industry, where a substantially reduced risk of HFS can be expected in comparison to the commercially available PEGylated Dox formulation Doxil. Furthermore, this study is providing a new and systematic approach to the development of new anticancer medication with reduced side effects and increased benefits for patients.

## Figures and Tables

**Table 1:** Composition of the soybean oil we used in our study.

Fatty Acids Composition for Soybean Oil	(%)
C 16:0 Palmitic acid	10.62
C 18:0 Stearic acid	2.81
C 18:1 Oleic acid	25.25
C 18:2 Linoleic acid	53.29
C 18:3 Linoleic acid	5.95
C 20:0 Arachidic acid	0.35
C 22:0 Behecid acid	0.70
Trans-isomer fatty acids	1.08

**Table 2:** Composition of the Mygliol 812 we used in our study.

Fatty Acids Composition for Mygliol 812	(%)
Caproic acid	0.10
Caprylic acid	56.3
Capric acid	43.1
Lauric acid	0.30
Myristic acid	0.10
